# Self-Concept and Self-Esteem, Determinants of Greater Life Satisfaction in Mountain and Climbing Technicians and Athletes

**DOI:** 10.3390/ejihpe13070088

**Published:** 2023-06-30

**Authors:** Laura Martín-Talavera, Óscar Gavín-Chocano, Guillermo Sanz-Junoy, David Molero

**Affiliations:** 1 Spanish Federation of Mountain Sports and Climbing, Floridablanca 84, 08015 Barcelona, Spain; secretaria.eeam@fedme.es (L.M.-T.); sistemagestion@fedme.es (G.S.-J.); 2 Department of Pedagogy, University of Jaén, 23071 Jaén, Spain; ogavin@ujaen.es

**Keywords:** life satisfaction, mountain sports, self-concept, self-esteem, sports and health

## Abstract

In recent decades, the exponential growth that sports practice in mountain and climbing disciplines have experienced has led to trying to find an explanation for the predisposition of those socio-emotional factors of these athletes that are considered in the search for sensations and the achievement of greater satisfaction with life. In this study, 4818 people with a sports license in the Spanish Federation of Mountain and Climbing Sports (FEDME) participated; 67.1% were men and 32.9% women. The mean age of the participants was 49.42 years (±11.9), between a range of 18 to 76 years. The following instruments were used: the Self-concept scale (AF5), the Rosenberg Self-esteem Questionnaire and the Life satisfaction scale (SWLS). The objective of this research was to study the enhancing effect of self-esteem between self-concept (physical, emotional and social) and life satisfaction. A reflective model of structural equations (PLS-SEM) was applied based on the proposed theoretical framework from an explanatory–predictive perspective. The results show the self-esteem determination coefficients [(*Q*^2^ = 0.141); (*R*^2^ = 0.302)] and life satisfaction [(*Q*^2^ = 0.243); (*R*^2^ = 0.342)] in the estimation of the model, indicating an adequate fit. Mountaineering and climbing sports can be very rewarding, but they can also be challenging and frustrating. Having a good self-concept and good self-esteem allows the athlete to enjoy achieving greater life satisfaction.

## 1. Introduction

Mountaineering and climbing are extreme sports that require a great deal of physical and mental skills, as well as a strong sense of self-concept and self-esteem. Mountain sports have a sporting environment with its own characteristics, which are different from other sport modalities and risk conditions in some cases and which may affect the profile of athletes physically and, above all, psychologically [1,2].

Currently, Spain has a large number of elite mountaineering and climbing athletes, and more and more people are interested in these sports. According to the Spanish Federation of Mountain and Climbing Sports (FEDME), in 2020, a total of 82,027 federation licenses were registered in Spain for the practice of mountain and climbing sports. This number increased exponentially after the pandemic that was suffered worldwide by COVID-19 by up to 107588. As the popularity of these outdoor activities has grown, it has become increasingly important to understand how emotional factors influence athletes’ performance and satisfaction [2]. The practice of physical activity is considered a way to develop personal potential and improve all facets of the human personality. The attitude towards this activity is a fundamental factor for the development of psychosocial aspects such as self-concept and self-esteem [3]. The positive development of these variables will contribute to life satisfaction, which in turn has a positive effect on self-concept [4].

### 1.1. Self-Concept

Self-concept refers to the perception that a person has about himself, that is, to the image or mental representation that he has of his characteristics, abilities, limitations, values and roles in different areas of his life, such as social, emotional, physical, academic, among others. This abstract image may be conditioned by past experiences, social interactions, cultural values and personal expectations [5,6]. Self-concept in mountaineering and climbing athletes is a key factor that can influence their performance and their general experience in practicing sports [7], despite the scarcity of information on the impact of mountain sports activities on self-concept [8]. A good self-concept in mountaineering and climbing athletes can increase their motivation, confidence, resilience and ability to overcome difficulties and achieve goals. On the contrary, a low self-concept can generate feelings of insecurity, anxiety, stress and a lack of confidence, which can negatively affect their performance and enjoyment in sports.

Self-concept is a generic term that includes several dimensions that include emotional, social, academic, physical and family dimensions [9]. Among these dimensions, the physical dimension (appearance, strength and sports ability, among others) has been positively linked to physical self-concept and motor coordination [10,11] with the participation in sports and physical activities [12]. It is related to health and physical conditions [13,14]. The model that we follow in this study [15] identifies five dimensions of self-concept: social, emotional, physical, familial and academic. This allows us to have a more complete vision of the different daily realities.

In recent years, several authors have studied the relationship between physical-sporting activities and self-concept. A study conducted by Lemoyne et al. [16] on students evidenced a positive relationship between the level of physical activity practice and self-concept. Along the same lines, different studies [14,17] have considered the relationship between the practice of physical activities and body image in young people, concluding that the most active subjects have a healthier body composition and are more satisfied with their body image, which reflects an improvement in their physical and general self-concept. When studies of self-concept in mountaineering and climbing athletes were examined, a significant relationship with self-esteem [18] and life satisfaction [19] was found.

### 1.2. Self-Esteem

Self-esteem refers to the assessment that a person has of himself in terms of his self-image. Healthy self-esteem is a critical factor in success in any area of life and is particularly important in mountain and climbing sports. Athletes need to have strong self-esteem to face the physical and mental challenges they face and to stay focused on their long-term goals. Bahaeloo-Horeh and Assari [8] demonstrated that participation in a single mountaineering program improved students’ sense of self-esteem. In turn, significant differences have been found in self-esteem values in favor of those people who practice mountain sports compared to those who do not practice mountain sports [20,21].

By relating self-esteem with other variables analyzed in this study, it has been verified that some subtypes of self-concept, such as social self-concept, have a negative relationship with self-esteem in athletes [6]. This aspect is relevant, since athletes with low self-esteem have difficulties in social adaptation [22,23]. By delving into the relationship between both variables, self-esteem and self-concept, cognitive, evaluative and affective indices that the individual makes of himself can be considered [24]. Self-esteem, as an assessment of oneself, involves a process of analysis and introspection about one’s own feelings, about the characteristics that define us and about abilities and achievements [25]. In relation to the possible differences that may appear based on gender at an early age, the evidence provided in different studies [26,27] revealed that the effect of perceived sports competence on global self-esteem is moderated by individual importance. Although men rated sports as more important than women, the results were not statistically significant.

### 1.3. Life Satisfaction

Life satisfaction is a concept related to subjective well-being and quality of life [28], and it can be evaluated in mountaineering and climbing athletes to understand its relationship with sports practice. Life satisfaction refers to the vision that one has of one’s life, that is, the judgment about one’s life [29]. This means that life satisfaction plays an important role in our lives and is affected by various areas: employment situation, age, health, among others). The psychological well-being caused by the multitude of contexts that we face, which intrinsically influence our life satisfaction, will depend on the way of facing and dealing with these situations through different personal, subjective and psychological dimensions [30].

When talking about the achievement of psychological well-being and, therefore, the life satisfaction that a person can achieve, Ryff and Singer [31] suggest a series of components that, according to Mosquera et al. [32], are necessary to achieve better levels of well-being, since each of the proposed dimensions works on a series of objectives required for the development of skills and abilities that generate this well-being. These are self-acceptance, positive relationships with others, autonomy, mastery of the environment, life purpose and personal growth. Frochot et al. [33] and Gavín-Chocano et al. [2] analyzed the satisfaction of practitioners of these mountain sports disciplines and the self-perceived well-being produced by this activity in mountain tourism contexts, finding relationships between them. It has also been shown that an outdoor sports activity in a natural environment improves self-esteem, and it is more restorative than a sports activity in an urban environment [34,35].

Some studies have explored the relationship between the practice of mountain sports and climbing and life satisfaction. A study by Fielding et al. [36] found that climbers showed greater life satisfaction than non-climbers. Likewise, in a study carried out by Valdés-Badilla et al. [37] found that the practice of mountain sports was positively related to life satisfaction. In addition, other studies have explored how specific factors in mountain sports and climbing can affect athletes’ life satisfaction. A study carried out by Aquino-Llinares and Gavala-González [38] found that the flow experience during climbing practice was positively related to athletes’ life satisfaction. In general, the practice of mountain and climbing sports seems to be related to greater life satisfaction in athletes, and some factors specific to the practice may influence this relationship. However, it is important to keep in mind that each athlete is unique and that results may vary depending on multiple personal and contextual factors.

### 1.4. Study Purpose and Hypotheses

**Figure 1:**
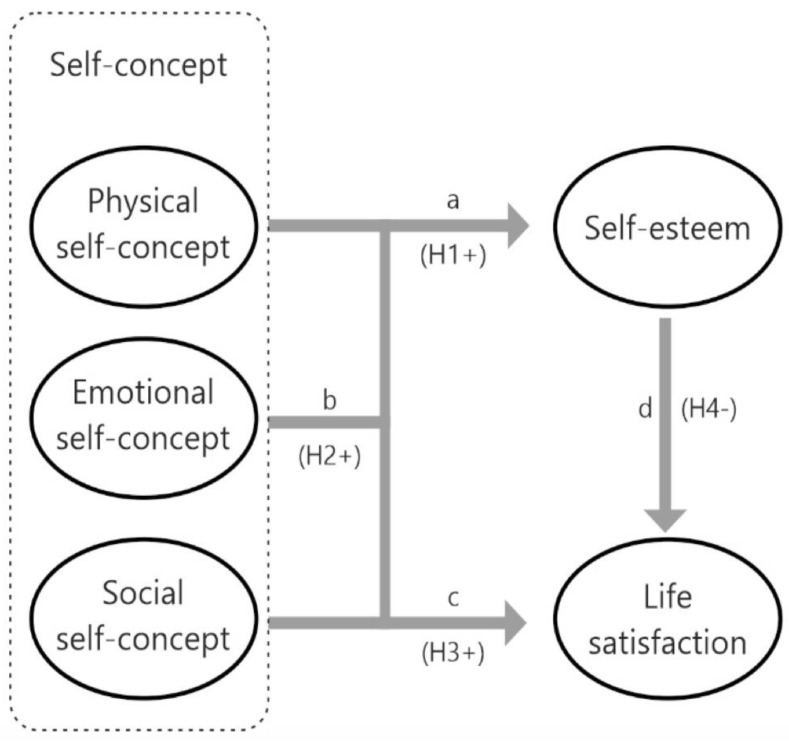
Proposed theoretical model.

Physical self-concept and self-esteem are two important variables that influence the performance of mountaineering and climbing athletes. A positive perception of physical abilities can increase athletes’ confidence and motivation [26], which in turn can improve their performance [25]. On the other hand, a negative perception of their body and physical abilities can decrease confidence and motivation, which can negatively affect performance [39]. That is, self-esteem can influence how athletes perceive and approach challenges and failures (See Figure 1). Positive self-esteem can help athletes cope with challenges and learn from failures, while low self-esteem can lead to feelings of insecurity and a lack of confidence [13].

**Hypothesis** **1 (H1):** *Physical self-concept will be related to self-esteem.*

Emotional self-concept refers to a person’s perception of their emotional abilities and their ability to manage their emotions and the emotions of others. Self-esteem, on the other hand, is the assessment and appreciation that a person has of himself and of his abilities and personal attributes [40]. Self-esteem and emotional self-concept in mountaineering and climbing athletes can be affected by external factors, such as social pressure and competition with other athletes. That is, an athlete with high self-esteem will probably have a positive emotional self-concept, even in extreme situations. On the other hand, an athlete with low self-esteem will probably have a negative emotional self-concept, due to the insecurity generated [41].

**Hypothesis** **2 (H2):** *Emotional self-concept will be related to self-esteem.*

Social self-concept refers to the personal perception of one’s own identity and competence in the social context, while self-esteem is a general assessment of oneself. In mountain sports and climbing, social self-concept and self-esteem can be influenced by a variety of factors, such as technical ability, the ability to work in a team, the ability to make important decisions in high-pressure situations, respect and the acceptance of climbing partners [6]. In other words, a mountain athlete with a high social self-concept may feel more confident and competent in their ability to work in a team and make important decisions, which in turn can improve their self-esteem [20].

**Hypothesis** **3 (H3):** *Social self-concept will be positively related to self-esteem.*

The relationship between self-esteem and life satisfaction is not linear and can be influenced by many factors, such as personality, life circumstances, physical image, social support and mental health [19,42]. Mountain athletes often compare their abilities and achievements with those of others, which can lead to feeling inadequate and low self-esteem. In the same way, different investigations that have analyzed the variables that mediate between satisfaction with life and self-esteem have concluded that emotional, physical and social self-concept [43] can condition the relationship between satisfaction with life and self-esteem. That is, mountain and climbing sports take place in dangerous environments, and the fear of failure or injury can affect self-confidence and self-esteem. Furthermore, the social pressure in the world of mountain sports can be intense, and not meeting expectations can negatively affect self-esteem.

**Hypothesis** **4 (H4):** *Self-esteem, as a mediating variable, will be negatively related to life satisfaction.*

## 2. Materials and Methods

### 2.1. Participants

The study participants were selected from a population of 107,588 people with a federation license in the Spanish Federation of Mountain and Climbing Sports (Federación Española de Deportes de Montaña y Escalada (FEDME)). For this, a probabilistic sampling was used (estimated error of 2% and a confidence level of 99%). The accepting sample consisted of 4818 technicians and athletes over 18 years of age, with a federation license in 2022. In relation to the distribution by gender, 2696 subjects were men (67.1%) and 1322 were women (32.9%). The mean age was 49.42 years old (±11.9).

### 2.2. Instruments

The Rosenberg Self-esteem Questionnaire [44,45] is made up of 10 items which assess the feeling of satisfaction that a person has about himself. A 5-point Likert scale (1 to 5 points, where 1 represented “Strongly disagree” and 5 represented “Strongly agree”) was used. The ranges to determine the level of self-esteem are the following: 30 to 40 points—high self-esteem, considered as a normal level of self-esteem; from 26 to 29 points—average self-esteem, indicating that there are no serious self-esteem problems, although it should be improved; and less than 25 points—low self-esteem, where there are significant self-esteem problems. In the present study, the reliability of the scale is as follows: *α* = 0.807 and *ω* = 0.814.

The Self-concept scale AF5 was employed [15]. The questionnaire consists of 30 items, formulated to be applied both individually and in groups. A 5-point Likert scale (1 to 5 points) was used, where 1 represented “Never” and 5 represented “Always”. Due to the characteristics of the sample, mountaineering and climbing athletes, three of the dimensions that make up the instrument were used: emotional self-concept, which refers to the subject’s perception of their emotional state and their responses to specific situations, with a certain degree of commitment and involvement in their daily life; physical self-concept, which refers to the perception that the subject has of his physical appearance and his physical condition; and social self-concept or the perception that the subject has of his performance in social relationships. In the present study, the reliability of the scale is as follows: emotional self-concept (*α* = 0.779 and *ω* = 0.786); physical self-concept (*α* = 0.743 and *ω* = 0.754); and social self-concept (*α* = 0.802 and *ω* = 0.810).

The Life satisfaction scale (SWLS) was created with the aim of measuring the level of satisfaction with life [46]. In its original study, this instrument demonstrated high internal consistency (*α* = 0.87) and a strong test–retest correlation coefficient (*r* = 0.82). The participants respond to a Likert scale with 7 options (1 to 7 points), according to the degree of agreement and disagreement with each item (where 1 represented “Strongly disagree” and 7 represented “Strongly agree”). The scale was adapted and validated in Spanish by Atienza et al. [47]. In the present study, the reliability of the scale is as follows: *α* = 0.884 and *ω* = 0.903.

### 2.3. Procedure

The ethical guidelines promoted by national and international regulations for conducting research with people were followed. All data were processed in accordance with EU Regulation 2016/679 of the European Parliament and of the Council, of 27 April 2016, both on Personal Data and Organic Law 3/2018, of 5 December 2018, regarding the guarantee of digital rights. Participants were assured that their responses would be kept anonymous and confidential, and that all information provided would be used for scientific purposes only. The instrument was administered individually through the Google^®^ platform (Google forms), between September and November 2022. The researchers explained to the participants the purpose of the research, as well as the guidelines for its proper compliance, requesting the voluntary collaboration of the students. The data were collected, and their quality were checked, ensuring at all times that the process complied with the ethical principles for research defined in the Declaration of Helsinki [48] and the standards of integrity in research of The European Code of Conduct for Research Integrity [49].

### 2.4. Data Analysis

Descriptive statistics (means and standard deviations) were obtained. Previously, the Hot-Deck multiple entry method was applied to reduce bias by preserving joint and marginal distributions [50], analyzing reliability (Cronbach’s alpha and McDonald’s omega coefficients) and validity through Confirmatory Factor Analysis (CFA) to verify the psychometric properties of the questionnaire and obtain the factor loadings of each item (See Supplementary Materials). The normality analysis was performed by contrasting the multivariate hypothesis, resulting in the normal distribution.

The analyses were carried out using the SPPS AMOS 25 program, the Jamovi software (The Jamovi Project) Version 1.2 and SmartPLS (version 3.3.6). Regarding the coefficients considered in this research, these were the *χ*^2^/*df* ratio, the Root Mean square Error of Approximation (RMSEA), the Comparative Fit Index (CFI) and the Tucker–Lewis Index (TLI). The external loads (rho_A or Dijkstra Henseler index), the degrees of the Composite Reliability Index (CRI or Dillon–Goldstein index) and the Average Variance Extracted (AVE) were calculated. The discriminant validity was obtained according to the criteria of Fornell and Larcker (Heterotrait–Monotrait HTMT).

The goodness of fit of the model was considered satisfactory when the TLI and CFI ≥ 0.95 and the RMSEA was close to 0.07 [51]. We used the Partial Least Squares (PLS) technique with an explanatory and predictive purpose of the dependent variables and types of relationships, direct and indirect [52]. Statistical significance required a 95% confidence level (significance *p* < 0.05).

## 3. Results

### Structural Model

**Figure 2:**
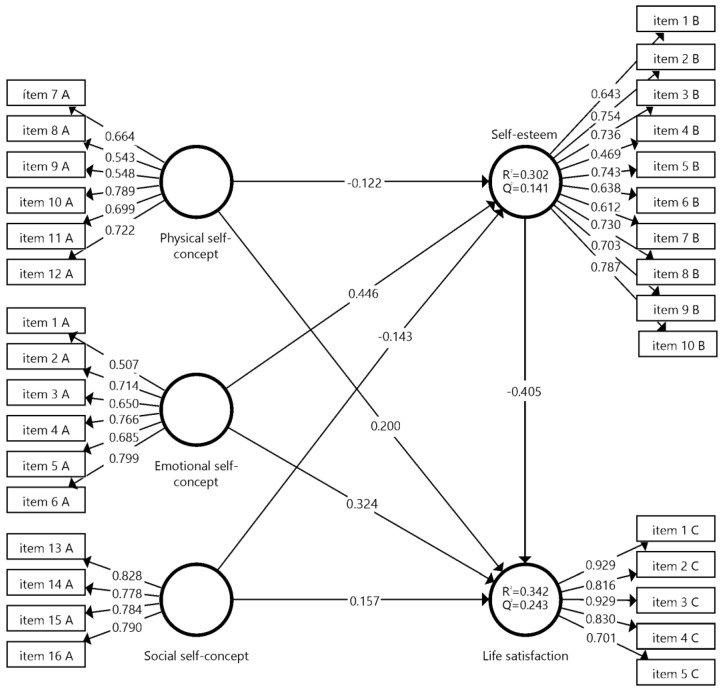
Proposed theoretical model.

Multicollinearity was verified between the antecedent variables of the endogenous constructs with the variance inflation factor (VIF) test by Hair et al. [53]. The VIF values should be close to 3 [53], corroborating that there were no collinearity problems. For the analysis of the structural model, Bootstrapping (5000 subsample) was performed, following the criteria of Henseler et al. [54], to verify standard errors and t statistics of the route coefficient with a confidence interval (95%) of the standardized regression coefficients (Figure 2). The established criteria were the coefficient of determination (*R*^2^) and cross-validated redundancy (*Q*^2^), as well as the trajectory between variables [53].

Table 1 shows Cronbach’s alpha (*α*), external loads (rho_A or Dijkstra–Henseler index) [54], the degrees of the Composite Reliability Index (CRI or Dillon–Goldstein’s index) and the average variance extracted (AVE) [55,56], where the values must be greater than 0.5 [57]. That is, a high value of the extracted average variance (AVE) will have a better representation of the load of the observable variable.

Discriminant validity (Table 2), according to the Fornell and Larcker [58] and Heterotrait–Monotrait (HTMT) criteria, involves comparing the square root of the AVE with the correlations. For satisfactory discriminant validity, the diagonal items (in bold) must be significantly higher than the off-diagonal items in the corresponding rows and columns [58]. Similarly, the Heterotrait–Monotrait (HTMT) correlation relationship shows the difference between the latent variable of each factor with respect to the others, indicating in bold the square root of the average variance extracted [59] and criteria that are met. All the HTMT values are below 0.85 and 0.90 [60].

The discriminant validity (Table 3) was analyzed through the analysis of the cross-loads of each of the latent variables with respect to the observed variables, with the loads being higher [61].

Table 4 shows the results of the hypothesis contrast, following the criteria of Hair et al. [53], to test the mediator effect in PLS-SEM. A t-test was obtained (values greater than 1.96 indicate the adequacy of the reflective model). The results showed the following: emotional self-concept -> life satisfaction (*β* = 0.324, *t* = 22.229, *p* < 0.001); emotional self-concept -> self-esteem (*β* = 0.446, *t* = 34.582, *p* < 0.001); physical self-concept -> life satisfaction (*β* = 0.200, *t* = 12.476, *p* < 0.001); social self-concept -> life satisfaction (*β* = 0.157, *t* = 10.136, *p* < 0.001) and, negatively, physical self-concept -> self-esteem (*β* = −0.122, *t* = 8.075, *p* < 0.001); self-esteem -> life satisfaction (*β* = −0.405, *t* = 27.811, *p* < 0.001); social self-concept -> self-esteem (*β* = −0.143, *t* = 9.386, *p* < 0.001), respectively, thus confirming the existence of mediation through the mediating variable self-esteem between emotional self-concept, physical self-concept, social self-concept and life satisfaction.

## 4. Discussion and Conclusions

The objective of this research was to establish the relationship between the dimensions of self-concept (emotional, physical and social) and life satisfaction, with self-esteem acting as a mediating variable in mountaineering and climbing athletes from the Spanish Federation of Mountaineering and Climbing Sports. A comprehensive model was formulated considering emotional self-concept, an inherent factor in emotional management and regulation in extreme situations; physical self-concept or personal perception of physical abilities in carrying out risky activities and of one’s own body [42]; social self-concept [20], which is related to the ability to interact with other athletes and to work as a team and, at the same time, with the ability to achieve goals and overcome challenges; as well as the feeling of being satisfied when in contact with nature [18]. We conclude that the proposed theoretical model has satisfactory predictive relevance of all its dimensions.

According to the first hypothesis (*H1*), physical self-concept was negatively related to self-esteem. Different studies corroborate these results, pointing out that high-level sports, such as mountain and climbing activities, often require a high level of physical ability and constant training, and athletes can be very critical of their own performance and physical appearance in comparison with others [8,62]. Similarly, injuries and accidents related to these sports can significantly affect the self-image and self-esteem of an athlete [40,63]. For example, if an athlete suffers an injury that prevents them from continuing their physical activity for a long time, they may feel frustrated and anxious, and their self-esteem may decrease due to the perception that their body is no longer capable of accomplishing what it could before. In this sense, a negative physical self-concept does not allow the athlete to perform a physical activity or sport successfully [64], affecting their psychological well-being and life satisfaction [65], in turn negatively affecting in the health of the person, especially in the case of high-level athletes.

Regarding the second hypothesis (*H2*), social self-concept was negatively related to self-esteem and positively related to life satisfaction. In this sense, some research has shown that the social self-concept in high-level athletes is negatively related to self-esteem [6]. That is, low levels of self-esteem have been related to less social adaptation and greater vulnerability in extreme situations [8,23]. This means that people who have a low social self-concept, that is, who perceive themselves negatively compared to others in their social environment, tend to have low self-esteem [20,22]. On the other hand, people who have a high social self-concept, that is to say, who perceive themselves positively compared to others in their social environment, tend to have high self-esteem. However, it is important to keep in mind that the relationship between social self-concept, self-esteem and life satisfaction can be complex and depends on various individual and contextual factors. In addition, social self-concept can change over time and be influenced by social experiences and events.

Regarding the third hypothesis (*H3*), social self-concept was positively related to self-esteem and satisfaction with life. Thus, the perception that a person has about himself in relation to his role and position within his social and cultural environment can be influenced by various factors, such as his level of ability and experience in these activities, belonging to a group of athletes, the image they project in their social environment, among others. Different investigations have related these variables, indicating that the perception of ability was positively related to self-esteem and life satisfaction and that social self-concept mediated this relationship [66,67]. Other studies related self-determined motivation and the satisfaction of basic psychological needs in climbers. The results indicated that self-determined motivation was positively related to the satisfaction of psychological needs, self-esteem and life satisfaction and that this relationship was mediated by the reduction of social anxiety [19,68].

Finally, the fourth hypothesis (*H4*) established the mediation of self-esteem between self-concept (physical, emotional and social) and life satisfaction. These results are consistent with different investigations that related the effect of self-esteem on life satisfaction [69], understanding the temporality of self-esteem in relation to people’s well-being. The relationship between self-esteem and life satisfaction can vary depending on the context and the individual characteristics of people, but in general, this research suggests that the two are positively related. That is, when self-esteem is high, life satisfaction also tends to be high [70,71]. In mountain and climbing athletes, low self-esteem affects the well-being of the person [72], being two interrelated constructs that mutually influence how people perceive their life. That is, low self-esteem can influence life satisfaction, while lower life satisfaction can negatively affect self-esteem [19].

### 4.1. Conclusions

The importance of carrying out research from the socio-emotional perspective in mountaineering and climbing athletes lies in the creation of strategies that, by addressing the variables that contribute to the increase in subjective well-being, foster higher levels of life satisfaction and promote a better physical, emotional and social self-concept. In this sense, it is essential to implement actions that take advantage of human potential for a constant process of personal growth, especially in athletes with a high level of risk in the development of their activity in nature.

It was verified that self-esteem has a negative potential in the subjective well-being of an individual, when it is related to the physical, emotional and social self-concept. Therefore, given the explanatory and predictive capacity of self-esteem on life satisfaction, interventions should be developed to strengthen self-esteem in this population, which will simultaneously improve levels of satisfaction with life, as evidenced in the results of this investigation.

An important conclusion is to analyze the practices that the performance of mountain sports has on mental and emotional health and not only on physical health. Some authors [8] have shown that a single practice of sports in the natural environment produces moderate beneficial effects on self-concept and self-esteem and that effects are produced continuously that remain visible over time. The mediation of self-esteem between self-concept (physical, emotional and social) and satisfaction with life has been demonstrated. The effect of self-esteem on life satisfaction and well-being is a reality.

The relationship between self-esteem and satisfaction with life can vary depending on the context and the individual characteristics of people, which is why it would be very useful in future studies to analyze the existence of significant differences in the variables considered among practitioners of mountain sports compared to people who do not practice these sports (mountain sports practitioners vs. non-practitioners), which some studies have analyzed [20,21]. Aspects such as those analyzed in this study have a high transfer to daily life and can influence greater satisfaction with life.

### 4.2. Limitations

However, this study has some limitations, among which the use of self-report measures stands out, which can lead to scores biased by social desirability, that is, the tendency of the participants to present themselves in a positive way. As possible future lines of research and based on the results of this study, it could be interesting to carry out longitudinal research or differentiate by mountain and climbing sports disciplines to observe significant differences or changes in the variables analyzed according to the sports trajectory of the participant subjects. Within the future lines of research, it will be necessary to compare the results obtained with those that can be obtained in other countries to establish if there are significant differences. Likewise, it will be necessary to analyze whether there are significant differences, at a statistical level, depending on the different disciplines of mountain sports.

## Figures and Tables

**Table 1:** Correlation weights, reliability estimates and convergent validity.

Variable	*α*	rho_A	Composite Reliability Index (CRI)	Average Variance Extracted (AVE)
Emotional self-concept	0.881	0.908	0.945	0.681
Life satisfaction	0.897	0.911	0.925	0.715
Physical self-concept	0.850	0.888	0.925	0.645
Self-esteem	0.873	0.883	0.898	0.673
Social self-concept	0.807	0.813	0.873	0.632

**Table 2:** Measurement model; discriminant validity.

**Fornell–Larcker Criterion**	**1**	**2**	**3**	**4**	**5**
1. Emotional self-concept	**0.693**				
2. Life satisfaction	−0.279	**0.845**			
3. Physical self-concept	−0.230	0.396	**0.667**		
4. Self-esteem	0.503	−0.508	−0.295	**0.687**	
5. Social self-concept	−0.207	0.374	0.491	−0.295	**0.795**
**Heterotrait–Monotrait Ratio**	**1**	**2**	**3**	**4**	**5**
Emotional self-concept					
Life satisfaction	0.325				
Physical self-concept	0.280	0.460			
Self-esteem	0.598	0.563	0.338		
Social self-concept	0.259	0.431	0.642	0.341	

**Table 3:** Cross-loads (latent and observable variables).

Variable	Emotional Self-Concept	Life Satisfaction	Physical Self-Concept	Self-Esteem	Social Self-Concept
ítem 1 A	**0.507**	−0.135	−0.056	0.234	0.002
ítem 2 A	**0.714**	−0.163	−0.088	0.342	−0.046
ítem 3 A	**0.650**	−0.168	−0.196	0.280	−0.210
ítem 4 A	**0.766**	−0.186	−0.175	0.359	−0.158
ítem 5 A	**685**	−0.211	−0.205	0.361	−0.229
ítem 6 A	**0.799**	−0.265	−0.205	0.462	−0.176
ítem 1 C	−0.265	**0.929**	0.376	−0.469	0.357
Ítem 2 C	−0.211	**0.816**	0.304	−0.444	0.277
ítem 3 C	−0.265	**0.929**	0.376	−0.470	0.358
ítem 4 C	−0.216	**0.830**	0.337	−0.413	0.308
ítem 5 C	−0.217	**0.701**	0.266	−0.339	0.268
ítem 7 A	−0.115	0.259	**0.664**	−0.207	0.312
ítem 8 A	−0.100	0.222	**0.543**	−0.137	0.470
ítem 9 A	−0.072	0.189	**0.548**	−0.129	0.268
ítem 10 A	−0.248	0.376	**0.789**	−0.299	0.337
ítem 11 A	−0.156	0.206	**0.699**	−0.148	0.314
ítem 12 A	−0.169	0.262	**0.722**	−0.188	0.306
ítem 1 B	0.323	−0.263	−0.132	**0.643**	−0.114
ítem 2 B	0.362	−0.354	−0.183	**0.754**	−0.193
ítem 3 B	0.469	−0.323	−0.161	**0.736**	−0.144
ítem 4 B	0.233	−0.275	−0.214	**0.469**	−0.210
ítem 5 B	0.304	−0.533	−0.313	**0.743**	−0.313
ítem 6 B	0.330	−0.261	−0.146	**0.638**	−0.162
ítem 7 B	0.286	−0.335	−0.221	**0.612**	−0.215
ítem 8 B	0.322	−0.423	−0.278	**0.730**	−0.273
ítem 9 B	0.411	−0.284	−0.146	**0.703**	−0.167
ítem 10 B	0.410	−0.356	−0.189	**0.787**	−0.193
ítem 13 A	−0.160	0.286	0.359	−0.211	**0.828**
ítem 14 A	−0.135	0.241	0.399	−0.197	**0.778**
ítem 15 A	−0.189	0.336	0.416	−0.275	**0.784**
ítem 16 A	−0.163	0.307	0.383	−0.240	**0.790**

**Table 4:** Path coefficient (standardized regression coefficient).

Relationship between Variables	Path Coefficient (*β*)	Standard Deviation	*t*-Statistic	*p*
Emotional self-concept -> Life satisfaction	0.324	0.014	22.229	***
Emotional self-concept -> Self-esteem	0.446	0.013	34.582	***
Physical self-concept -> Life satisfaction	0.200	0.016	12.476	***
Physical self-concept -> Self-esteem	−0.122	0.015	8.075	***
Self-esteem -> Life satisfaction	−0.405	0.015	27.811	***
Social self-concept -> Life satisfaction	0.157	0.015	10.136	***
Social self-concept -> Self-esteem	−0.143	0.015	9.386	***

*Note*. ***: *p* < 0.001.

## Data Availability

Data are unavailable due to privacy or ethical restrictions. A statement is still required.

## Supplementary Materials

The following supporting information can be downloaded at: https://www.mdpi.com/article/10.3390/ejihpe13070088/s1, Table S1: Factor loadings Rosenberg Self-esteem scale; Table S2: Factor loadings Self-concept scale; Table S3: Factor loadings Life satisfaction scale.
