# Supplementary material for: Self-Concept and Self-Esteem, Determinants of Greater Life Satisfaction in Mountain and Climbing Technicians and Athletes
Source: Eur J Investig Health Psychol Educ. 2023 Jun 30;13(7):1188–201. doi: 10.3390/ejihpe13070088 (PMC10378547; doi:10.3390/ejihpe13070088)
Supplement: Supplementary file 1 [file ejihpe-13-00088-s001.zip › ejihpe-2453755-supplementary.pdf]

European Journal of
Investigation in Health, Psychology and Education

MDPI

Supplementary Materials

Self-Concept and Self-Esteem, Determinants of Greater Life Satisfaction in Mountain and Climbing Technicians and Athletes

Supplementary Materials

Laura Martín-Talavera [1], Óscar Gavín-Chocano [2], Guillermo Sanz-Junoy [1] and David Molero [2,*]

[1] Spanish Federation of Mountain Sports and Climbing, Floridablanca 84, 08015 Barcelona, Spain; secretaria.eeam@fedme.es (L.M.-T.); sistemagestion@fedme.es (G.S.-J.)
[2] Department of Pedagogy, University of Jaén, 23071 Jaén, Spain; ogavin@ujaen.es (Ó.G.-C.); dmolero@ujaen.es (D.M.)
* Correspondence: dmolero@ujaen.es; Tel.: +34 953 213436

Supplementary Materials

The assumptions of multicollinearity, homogeneity and homoscedasticity were analyzed to verify that the resulting distribution met the criteria of dependency between variables. Based on the data obtained with each of the instruments (see Table 1 to Table 3 of the Supplementary Materials), a Confirmatory Factor Analysis (CFA) was performed to verify the validity and internal structure of each item.

Table 1. Factor loadings; Rosenberg Self-esteem scale (Supplementary Materials).

Factor	Indicator	α	ω	Estimate	SE	Z	p	β	AVE	CR
Self-esteem	item 1	.861	.866	.688	.0148	46.4	< .001	.632	.419	.881
	item 2	.851	.857	.795	.0138	57.4	< .001	.740		
	item 3	.854	.859	.808	.0146	55.4	< .001	.722		
	item 4	.877	.880	.366	.0147	24.9	< .001	.368		
	item 5	.857	.861	.545	.0112	48.7	< .001	.657		
	item 6	.863	.867	.706	.0157	44.9	< .001	.614		
	item 7	.866	.871	.459	.0126	36.3	< .001	.517		
	item 8	.857	.862	.588	.0122	48.3	< .001	.653		
	item 9	.856	.862	.714	.0138	51.9	< .001	.688		
	item 10	.849	.854	.787	.0127	62.0	< .001	.782		

Note. Rosenberg Self-esteem scale: SE: Standardized error; Z: Z-value in the estimate; p: p-value of Z estimate; β: Standardized estimate; AVE: Average Variance Extracted; CR: Composite Reliability.

Rosenberg Self-esteem scale. The factor loadings for the items of this scale presented an adequate fit [49], $\chi 2/df = 3.639$, with CFI = 0.981, SRMR = .063 and RMSEA = .077 (See Table 2 of the Supplementary Materials). The reliability of this scale was Cronbach's α = .807 and McDonald's ω = .914.

Table 2. Factor loadings; Self-concept scale.

Factor	Indicator	*A*	*ω*	Estimate	SE	Z	*p*	*β*	*AVE*	CR
Emotional self-concept	item 1	.781	.787	.375	0.01442	26.0	< .001	.402	.485	.785
	item 2	.737	.747	.598	0.01356	44.1	< .001	.636		
	item 3	.749	.760	.516	0.01310	39.4	< .001	.577		
	item 4	.725	.732	.658	0.01258	52.3	< .001	.727		
	item 5	.755	.761	.613	0.01507	40.7	< .001	.595		
	item 6	.727	.733	.643	0.01224	52.6	< .001	.729		
Physical self-concept	item 7	.711	.725	.398	0.01076	37.0	< .001	.555	.443	.756
	item 8	.738	.746	.443	0.01385	31.9	< .001	.497		
	item 9	.734	.741	.468	0.01530	30.6	< .001	.473		
	item 10	.690	.702	.568	.01236	46.0	< .001	.666		
	item 11	.685	.698	.528	.01173	45.0	< .001	.654		
	item 12	.683	.702	.559	.01279	43.7	< .001	.646		
Social self-concept	item 13	.709	.727	.725	.01219	59.5	< .001	.793	.517	.811
	item 14	.754	.762	.497	.00946	52.6	< .001	.721		
	item 15	.780	.794	.505	.01109	45.6	< .001	.647		
	item 16	.758	0770	.690	.01334	51.7	< .001	.711		

Note. Self-concept scale: SE: Standardized error; *Z*: Z-value in the estimate; *p*: *p*-value of *Z* estimate; *β*: Standardized estimate; AVE: Average Variance Extracted; CR: Composite Reliability.

Self-concept scale AF5 [12]. The factor loadings for the items of this scale also presented adequate fit values, $\chi 2/df$ = 2.937, with CFI = 0.970, SRMR = .056 and RMSEA = .077 (see Table 3 of the Supplementary Materials). The reliability of this scale was emotional self-concept (α=.779 and ω=.786); physical self-concept (α=.743 and ω=.754); and social self-concept (α=.802 and ω=.810).

Table 3. Factor loadings; Life satisfaction scale.

Factor	Indicator	*α*	*ω*	Estimate	SE	*Z*	*p*	*β*	*AVE*	CR
Life satisfaction	item 1	.832	.853	1.174	.0120	98.1	< .001	.987	.584	.870
	item 2	.864	.895	0.751	.0145	51.6	< .001	.659		
	item 3	.833	.853	1.173	.0120	98.1	< .001	.893		
	item 4	.857	.892	0.767	.0148	52.0	< .001	.662		
	item 5	.910	.915	0.867	.0218	39.7	< .001	.531		

Note. Life satisfaction scale: SE: Standardized error; *Z*: Z-value in the estimate; *p*: *p*-value of *Z* estimate; *β*: Standardized estimate; AVE: Average Variance Extracted; CR: Composite Reliability.

Life satisfaction scale. In this third scale, the adjustment was also adjusted based on the values of the factor loadings of the items, $\chi 2/df$ = 2.484, with CFI = 0.971, SRMR = .059 and RMSEA = .079. The reliability of this scale was Cronbach's α = .884 and McDonald's ω = .903.

.
